# Recommendations for design of a mobile application to support management of anxiety and depression among Black American women

**DOI:** 10.3389/fdgth.2022.1028408

**Published:** 2022-12-23

**Authors:** Terika McCall, Megan Threats, Malvika Pillai, Adnan Lakdawala, Clinton S. Bolton

**Affiliations:** ^1^ Division of Health Informatics, Department of Biostatistics, Yale School of Public Health, New Haven, CT, United States; ^2^ Center for Medical Informatics, Yale School of Medicine, New Haven, CT, United States; ^3^ Center for Interdisciplinary Research on AIDS (CIRA), Yale School of Public Health, New Haven, CT, United States; ^4^ School of Information, University of Michigan, Ann Arbor, MI, United States; ^5^ Carolina Health Informatics Program, University of North Carolina at Chapel Hill, Chapel Hill, NC, United States; ^6^ Department of Medicine, Stanford University, Stanford, CA, United States; ^7^ Elimu Informatics, El Cerrito, CA, United States; ^8^ Rex Bariatric Specialists, Rex/UNC Hospitals, Raleigh, NC, United States

**Keywords:** African Americans, women, mental health, anxiety, depression, telemedicine, mHealth, digital health

## Abstract

Black American women experience adverse health outcomes due to anxiety and depression. They face systemic barriers to accessing culturally appropriate mental health care leading to the underutilization of mental health services and resources. Mobile technology can be leveraged to increase access to culturally relevant resources, however, the specific needs and preferences that Black women feel are useful in an app to support management of anxiety and depression are rarely reflected in existing digital health tools. This study aims to assess what types of content, features, and important considerations should be included in the design of a mobile app tailored to support management of anxiety and depression among Black women. Focus groups were conducted with 20 women (mean age 36.6 years, SD 17.8 years), with 5 participants per group. Focus groups were led by a moderator, with notetaker present, using an interview guide to discuss topics, such as participants' attitudes and perceptions towards mental health and use of mental health services, and content, features, and concerns for design of a mobile app to support management of anxiety and depression. Descriptive qualitative content analysis was conducted. Recommendations for content were either informational (e.g., information to find a Black woman therapist) or inspirational (e.g., encouraging stories about overcoming adversity). Suggested features allow users to monitor their progress, practice healthy coping techniques, and connect with others. The importance of feeling “a sense of community” was emphasized. Transparency about who created and owns the app, and how users' data will be used and protected was recommended to establish trust. The findings from this study were consistent with previous literature which highlighted the need for educational, psychotherapy, and personal development components for mental health apps. There has been exponential growth in the digital mental health space due to the COVID-19 pandemic; however, a *one-size-fits-all* approach may lead to more options but continued disparity in receiving mental health care. Designing a mental health app for and with Black women may help to advance digital health equity by providing a tool that addresses their specific needs and preferences, and increase engagement.

## Introduction

In 2020, an estimated 1 in 4 non-Hispanic Black women in the U.S. experienced mental illness (1). Anxiety and mood disorders are the most common mental health conditions among Black women (2). Despite the need, there are many barriers that attribute to Black women significantly underutilizing mental health services (14.3%) (1). Data from the 2020 *National Survey on Drug Use and Health* showed that 32% of non-Hispanic Black women who reported experiencing mental illness in the past year did not receive mental health treatment during that time (1).

A survey of nearly 400 Black women (representing 36 States) revealed the most common reasons for not seeking mental health treatment or counseling when needed were due to cost, not knowing where to access services, lack of time, and social stigma (3). Taking into consideration the aforementioned rates of mental illness, the effects of structural gendered racism, and the disproportionate impact the COVID-19 pandemic had on the Black community (4), we now have a potential mental health crisis within a group that is already overburdened and underserved. The use of mobile applications (apps) to deliver mental health services and resources may help diminish barriers and improve linkage and retention of Black women receiving mental health care. However, the application of technology for health care often takes longer to reach traditionally underserved populations due to systemic racism (e.g., lack of investment in communities of color), systemic oppression, and underrepresentation in health research (5).

Results from previous studies using mobile apps to deliver mental health interventions to reduce anxiety or depressive symptoms revealed that participants experienced a significant reduction in symptoms post-intervention (6, 7). However, most of the studies conducted have no to low representation of Black American women participants, which may affect the generalizability of the effectiveness for this group. In general, Black women are comfortable with participating in mobile health (mHealth) research and interventions (8, 9), and at least 80% of Black women own smartphones (10). This presents a great opportunity to use mobile apps to help reduce the disparity in the use of mental health services and improve health outcomes for Black women.

While there are commercial apps available that are geared toward women of color, most are focused on overall mental wellness and not specifically on reducing symptoms of anxiety and depression. The purpose of this study is to determine the content, features, and considerations for design of a mobile app tailored to support management of anxiety and depression among Black women. Providing a basis for factors to consider in development to increase usefulness, effectiveness, and user engagement.

## Materials and methods

### Recruitment

The study was exempted from full review by the University of North Carolina at Chapel Hill (UNC) Institutional Review Board (IRB # 19-2548). Participants were recruited *via* the Attitudes Toward Seeking Mental Health Services and Use of Mobile Technology Survey (3) (i.e., survey respondents indicated if they would like to be contacted about an opportunity to participate in the focus group), posts on social media (e.g., Twitter), and flyers posted in the Durham and Chapel Hill, North Carolina communities inviting women (18 years or older) who identified as Black or African American or multiracial (i.e., Black or African American and another race) and had a history of anxiety or depression to participate in the study. However, study participation did not require a clinical diagnosis of an anxiety or depressive disorder. Participants received a screening call to assess eligibility. Results from a study by Guest and colleagues revealed that more than 80% of all themes are discoverable within two to three focus groups, and 90% of themes could be discovered within three to six focus groups (11). Therefore, four focus group sessions were conducted. Each session was capped at five participants to allow for all participants to fully engage in the discussions.

### Procedures

In January 2020, the focus group sessions were held in Durham, NC in a private meeting room at a county library and at the University of North Carolina at Chapel Hill. TM (health informatics and mental health disparities researcher) moderated each session, and a notetaker (MT or MP, health informatics researchers) was present to take notes on body language, non-verbal cues, and emerging themes. The moderator met with the notetakers to discuss non-verbal cues to note (e.g., hesitation to speak). Additionally, one of the notetakers (MT) was trained through a qualitative data collection course *via* the Odum Institute for Research in Social Science at UNC, and through previous note-taking while conducting interviews and focus groups with Black and Latinx women as a graduate research assistant. Prior to the start of the sessions, the moderator went through consent forms with participants and obtained their signatures. In addition, participants were informed that the study would last approximately 1 h to 1 h 15 min. Participants were advised that the session is confidential and that anything shared in the room should not be discussed outside of the session. The sessions were recorded using an audio recorder. Each participant received a $25 gift card and an information sheet with mental health resources (e.g., therapist directory, Therapy for Black Girls podcast) to use personally or share with others. The moderator and notetaker debriefed after each session. All participants were given a study participant ID (PID). The transcripts were de-identified, with all possible identifiers removed and names replaced with PID. All data was securely stored digitally (HIPAA compliant storage) or physical locations (locked file drawer and office).

### Measures

A focus group interview guide was developed to help the moderator facilitate the discussions (Appendix A). The interview guide was developed in consultation with a qualitative data expert at Odum Institute for Research in Social Science at UNC to better tailor the questions and improve the flow of the discussion. Characteristics of study participants, such as age, race, and education were collected during the screening call to assess eligibility. The first part of the focus group session was used to ask questions about the following topics: (a) past and current causes of stress, anxiety and/or depression, and coping skills used; (b) attitudes and perceptions towards mental health and receiving mental health treatment; and (c) time in their life they felt anxious or depressed, and what type of support and/or resources would have been helpful to have access to during that time. The focus of this paper is on the last half of the session where participants were asked questions about: (a) personal use of mental health and wellness apps; (b) what they liked most and least about those apps; (c) key topics that should be covered in the content of a mobile app designed to help Black women manage anxiety and depression; (d) preferred resources and features; (e) concerns about use of the app; and (f) potential facilitators and barriers to use of the app.

### Data processing and analysis

The waveform audio files (.wav) were professionally transcribed and imported into NVivo 12 software for analysis (12). TM (focus group moderator) compared the transcripts to the audio files to confirm accuracy. TM and CB (licensed clinical mental health counselor) served on the analytic team, and independently coded the first interview using a grounded theory approach to inductively produce an initial list of the emerging topics from focus group feedback on questions regarding content, features, daily active use, and trust (i.e., concerns about using the app). Consensus was reached between the coders on the reoccurring topics. The remaining three focus group interview transcripts were coded independently using the agreed upon themes/subthemes, then TM and CB convened to discuss their analyses and explore the data through questions and comparisons of all four interview transcripts for additional themes/subthemes. The coding procedures facilitated a collaborative analytic process (13).

Descriptive qualitative content analysis was conducted for all focus group transcripts. “Qualitative content analysis is a dynamic form of analysis of verbal and visual data that is oriented toward summarizing the informational contents of that data” (14). Furthermore, a pragmatic qualitative research approach was employed to offer a “comprehensive summary of events in the everyday terms of those events” (14) for responses to questions that describe personal experiences or opinions. For example, a participant may describe past experiences using mental health apps and events that caused them to be concerned with using the apps. Thematic saturation was reached, as there was no new useful information produced after analysis of all transcripts.

## Results

**Figure 1:**
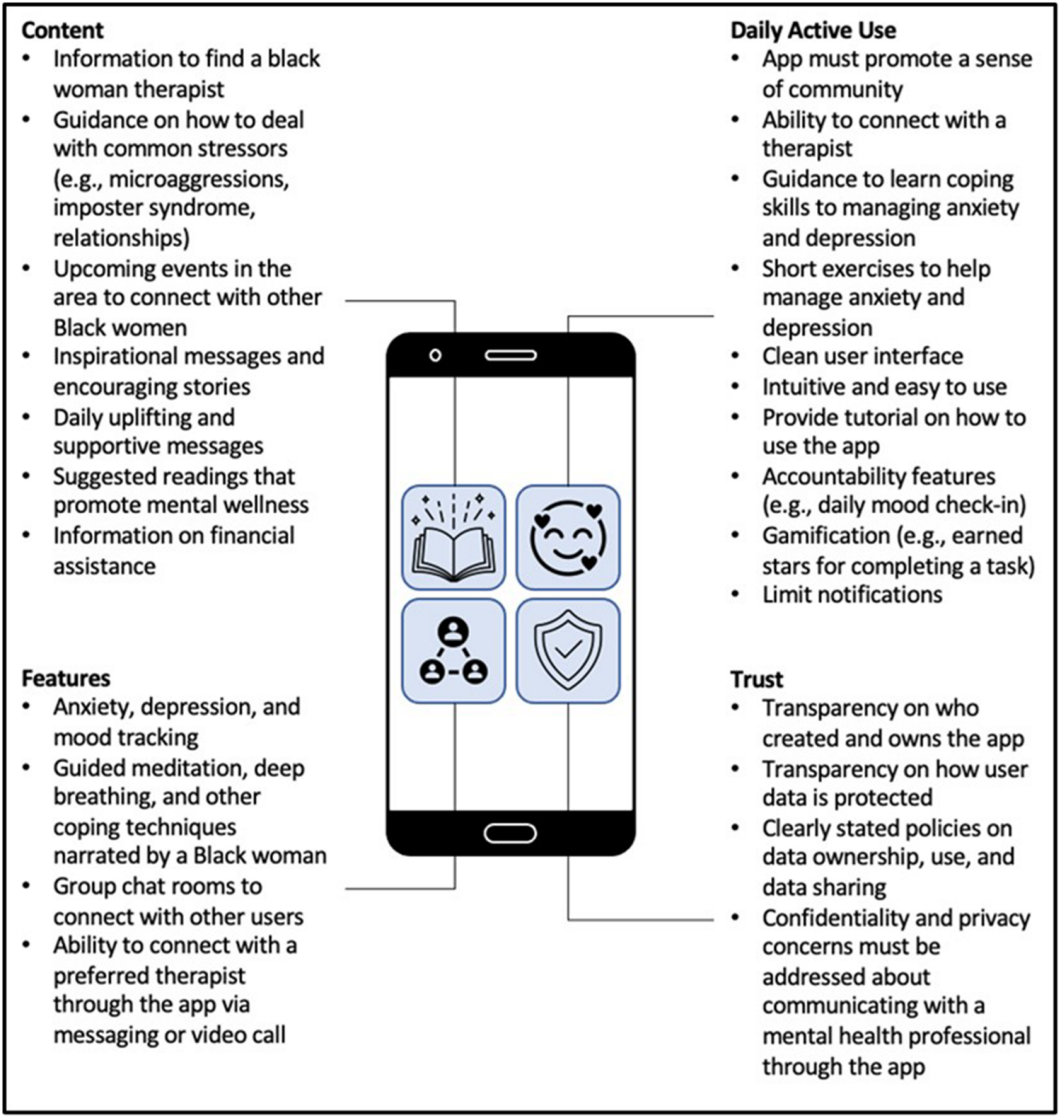
User-centered recommendations for the design of a mobile app to support management of anxiety and depression among Black American women.

Twenty participants attended the focus group sessions (*n* = 4). Each focus group consisted of five participants. Participants could only attend one focus group session (approximately 1 h to 1 h and 15 min). Study participants ranged in age from 21 to 79 years (mean age of 36.6 ± SD 17.8 years), and all identified as female and either Black/African American or multiracial. Most participants obtained a Bachelor's degree or higher (19/20, 95%). To engage a wide range of women and facilitate intergenerational discussion, half of the focus groups were mixed to include at least two participants age 50 years or older. For comparison, one focus group consisted of women under the age of 30 years old. Consensus was reached between the coders (TM and CB) on the following reoccurring topics: (a) content – informational, inspirational; (b) features – monitor progress, coping techniques, connect with others; (c) daily active use – value, ease of use, engagement, sense of community; and (d) trust – security and privacy, ownership, data use/sharing. Focus group participants shared that in the past they primarily used mental health and wellness apps that had the following features: meditation, mood tracking, calorie intake and activity monitoring, and deep breathing exercises. In addition, the use of music apps and podcasts (e.g., Therapy for Black Girls) were popular. Inspirational messages on social media apps were mentioned as also being beneficial to mental wellness. Figure 1 highlights the user-centered recommendations for the design of a mobile app to support management of anxiety and depression among Black women. Supporting quotes for the recommendations are presented in Table 1.

**Table 1:** Supporting quotes for recommendations on design of a mobile app to support management of anxiety and depression among Black women.

Themes	Examples
Content	
Informational	“…find Black women therapists in the area, with also listing what insurances they take, their hours, all that good stuff, because, again, those barriers. People are just like, where are these people?” [Speaker 4, Group 2, 23 years old] “If they might have a group somewhere that you could participate in that's in the area.” [Speaker 4, Group 4, 62 years old] “Definitely a resource about imposter syndrome because I feel like pretty much every Black woman experiences it but don't have the language to say what they're experiencing about it.” [Speaker 5, Group 1, 23 years old]
Inspirational	“I kind of would like if there was a spotlight. If there was like, “Oh this person…hear her story, she's going through this.” Then you can be like, ‘Oh I relate to that. I wonder how she deals with anxiety?’ Then you can be like, ‘Oh that worked for her, let me try that.” [Speaker 1, Group 3, 21 years old] “I guess the app [could] have some kind of a narrative depending on what the situation is, what's causing you to be anxious. Something that you could read that might help you.” [Speaker 1, Group 4, 66 years old]
Features	
Monitor progress	“If you could have a history in there and then you could look back and see when the last time was that you had this anxiety and what helped when you have this anxiety attack or whatever. You don't just end it with what you see. You can write down what helped and then you can go back to that.” [Speaker 3, Group 1, 63 years old] “If the notifications … Sorry. If the popup notifications could also be in a Black woman auntie voice, like, “Hey baby, have you … ” [Speaker 4, Group 2, 23 years old]
Coping techniques	“I would like the meditation still, but it'd be nice if it was a Black woman's voice. They're always Australian white men.” [Speaker 1, Group 2, 27 years old] “I would say a section on meditation and breathing. I know for me sometimes I hold my breath but just that reminder, Oh Hey breathe. But, but yeah definitely a section on teaching, like understanding meditation and, how that just taking a break during the day can help relieve anxiety.” [Speaker 3, Group 3, 34 years old]
Connect with others	“I also think, if there were to be a mental health app, maybe … There was one, I didn't use it, but they had, it's kind of like Slack where they have channels of like things you could talk about with people. So, I feel like if we're making one for Black women, one that I guess, has a group chat feature to be able to just actually talk to people because I feel like talking to people is a good way to build that rapport and like you said, talk to people. We depend on each other. So having a space, a shared space to be able to do that would be good.” [Speaker 5, Group 1, 23 years old] “I would love to be able to text someone through the app. Like a therapist or something. I find myself, sometimes I don't want to initially talk to anybody but sometimes I just need somebody to unload something on really quickly, who again, is just super far removed so I can move on with my day or whatever or evening, whatever time it is that I need that.” [Speaker 2, Group 1, 36 years old]
Daily active use	
Value	“I'd use things regularly that are helping me…Something about the app has got to be attractive enough to the individual that they'll want to continue using it.” [Speaker 1, Group 4, 66 years old] “I feel like knowing it's geared towards Black women that's using it, for me that would be incentive because I think about all the things I think or felt, I think for me, I'm at a point in my life where it's really important for me to see Black views, Black women at whatever term.” [Speaker 2, Group 1, 36 years old]
Ease of use	“It has to be user friendly for all ages.” [Speaker 4, Group 3, 29 years old] “I wouldn't want my app to be clunky. That might not be the right term for it, but I want to go to this app specifically for the mental health stuff, and not be bombarded with other things too. I just want it to be simple, and I can just cleanly use it.” [Speaker 1, Group 2, 27 years old]
Engagement	“Although it would be a superficial incentive, I feel just like apps that keep track or give you stars or anything that I guess signifies that you've completed something in itself can be validating to people, especially good habit building and stuff like that.” [Speaker 5, Group 1, 23 years old] I do like the idea of a thought journal, just a place to put what you're thinking and get it out there. [Speaker 3, Group 4, 29 years old]
Sense of community	“Getting together with my sisters and talking.” [Speaker 2, Group 3, 61 years old] “Just the idea that I could go to [the app] and maybe find a person that I could talk to.” [Speaker 4, Group 4, 62 years old]
Trust	
Security and privacy	“I think security is a big one for me just because you hear about all the hacks and everything. Even with…some telehealth apps and then I'm kind of skeptical of those too just because of the idea of security.” [Speaker 1, Group 1, 29 years old] “Would it be easy for somebody else to access your information once you're on there? That'd be another thing you have to worry about.” [Speaker 4, Group 4, 62 years old]
Ownership	“I was going to say who makes it, I know it's for Black woman, but who's making it, who's informing it? Because I feel like the fact that it is for us, it should be made by us from top to bottom. But I know that's not always possible because capitalism.” [Speaker 5, Group 1, 23 years old] “I worry about who owns the app. Right? Because we still live in a capitalistic society and so, depending on who's funding it, how that interacts with other apps. I don't want to be on my Facebook and then something that I talked about with or was texting with a therapist about pops up in an ad on Facebook. I'll be super salty if that happens. Right?” [Speaker 2, Group 1, 36 years old]
Data use/sharing	“I think that, and this is the old lady in me. I was watching the news on the treadmill last night, two nights ago, and they were talking about how these big companies on these dating apps are selling data, and so I would be worried about who has control over the data that's happening in the app, and what are they going to do with it now that they have all this information about Black women? That would scare me, about a non-Black woman or some type of corporation, and I don't know the legalese of sharing data, but that would be my biggest thing.” [Speaker 1, Group 2, 27 years old] “Tuskegee, Henrietta…I mean.” [Speaker 3, Group 2, 29 years old]

### Content

Many suggestions were given on the content that should be included in the app. Recommendations were either informational or inspirational. Participants stated they would like information about how to find a Black woman therapist in their area, how to deal with common stressors (e.g., imposter syndrome, microaggressions, family), financial assistance, and events in the area to connect with other Black women. Regarding content about finding a Black woman therapist, a participant voiced she would like to be able to:

 “…find Black women therapists in the area, with also listing what insurances they take, their hours, all that good stuff, because, again, those barriers. People are just like, where are these people?” [Speaker 4, Group 2, 23 years old]

Topics such as how to overcome imposter syndrome were desired:

“Definitely a resource about imposter syndrome because I feel like pretty much every Black woman experiences it but don't have the language to say what they're experiencing about it.”

[Speaker 5, Group 1, 23 years old]

Inspirational messages and encouraging stories about how others overcame adversity were also desired. Positive messages about self-esteem, as well as uplifting and supportive messages, were recommended. Participants also mentioned including suggested readings that promote mental wellness. One participant stated the following about encouraging stories:

“I kind of would like if there was a spotlight. If there was like, ‘Oh this person…hear her story, she's going through this.’ Then you can be like, ‘Oh I relate to that. I wonder how she deals with anxiety?’ Then you can be like, ‘Oh that worked for her, let me try that.’” [Speaker 1, Group 3, 21 years old]

In summary, the focus group participants recommend the app be informative, encouraging, and facilitate connection with resources.

### Features

The recommended app features would allow users to monitor their progress, practice coping techniques, and connect with others. Participants suggested that the app have features to track anxiety, depression, and mood. For example, a participant voiced she would like:

“If you could have a history in there and then you could look back and see when the last time was that you had this anxiety and what helped when you have this anxiety attack or whatever. You don't just end it with what you see. You can write down what helped and then you can go back to that.” [Speaker 3, Group 1, 63 years old]

They also recommended guided meditation, deep breathing, and other coping techniques narrated by a Black woman. Participants stated that most meditation apps used a British or Australian voice to narrate, and they desired to hear what they described as a “Black auntie voice” (i.e., the caring voice of a middle-aged Black woman). A participant stated:

“I would like the meditation still, but it'd be nice if it was a Black woman's voice. They're always Australian white men.” [Speaker 1, Group 2, 27 years old]

Another participant echoed:

“I just wasn't fond of the voices and the meditations themselves of certain apps.” [Speaker 5, Group 1, 23 years old]

There was also a desire to have the notifications written in the “Black auntie voice”:

“If the notifications … Sorry. If the popup notifications could also be in a Black woman auntie voice, like, “Hey baby, have you … ” [Speaker 4, Group 2, 23 years old]

Lastly, they recommended having group chat rooms to connect with other users, and the ability to connect with therapists through the app *via* messaging or video call. Regarding group chat rooms, one participant stated:

“I also think, if there were to be a mental health app, maybe … There was one, I didn't use it, but they had, it's kind of like Slack where they have channels of like things you could talk about with people. So, I feel like if we're making one for Black women, one that I guess, has a group chat feature to be able to just actually talk to people because I feel like talking to people is a good way to build that rapport and like you said, talk to people. We depend on each other. So, having a space, a shared space to be able to do that would be good.” [Speaker 5, Group 1, 23 years old]

Regarding connecting with therapists, a participant voiced:

“I would love to be able to text someone through the app. Like a therapist or something. I find myself, sometimes I don't want to initially talk to anybody but sometimes I just need somebody to unload something on really quickly, who again, is just super far removed so I can move on with my day or whatever or evening, whatever time it is that I need that.”

[Speaker 2, Group 1, 36 years old]

### Daily active use

Participants voiced that they would be more likely to use the app regularly if they found value in it and the app was easy to use. Specifically, participants emphasized the importance of feeling “a sense of community” when using the app, being able to connect with a therapist, and managing anxiety and depression through learning coping skills. One participant stated the following:

“I'd use things regularly that are helping me…Something about app has got to be attractive enough to the individual that they'll want to continue using it.” [Speaker 1, Group 4, 66 years old]

The user interface should also be “clean” (i.e., well-organized and uncluttered), and the app must be intuitive and easy to use. Having an accountability feature was also recommended (e.g., daily mood check-in). Gamification of the app was suggested to give the user a sense of accomplishment and make the app “stickier” (i.e., engaging and used regularly). Participants voiced that if the app is too cumbersome, there are too many notifications, or if the exercises take too long to complete, it will discourage use of the app. Regarding providing some type of incentive to use the app, one participant stated:

“Although it would be a superficial incentive, I feel just like apps that keep track or give you stars or anything that I guess signifies that you've completed something in itself can be validating to people, especially good habit building and stuff like that.” [Speaker 5, Group 1, 23 years old]

Overall, the focus group participants were excited about the idea of an app tailored to help them manage anxiety and depression and expressed interest in using it regularly.

### Trust

Most of the participants' concerns were about security and privacy. There was concern that the app could be hacked, and their data disclosed. Furthermore, participants had apprehension about who would own the app and their data sharing policies. There have been many data breaches in the news that concerned participants, and they were also aware that many apps sell user data. The primary concern was that the data would be used to harm them personally or Black women in general. A participant voiced her concern stating:

“I think that, and this is the old lady in me. I was watching the news on the treadmill last night, two nights ago, and they were talking about how these big companies on these dating apps are selling data, and so I would be worried about who has control over the data that's happening in the app, and what are they going to do with it now that they have all this information about Black women? That would scare me, about a non-Black woman or some type of corporation, and I don't know the legalese of sharing data, but that would be my biggest thing.” [Speaker 1, Group 2, 27 years old]

### Generational differences

Overall, focus group participants echoed similar sentiments regarding their needs and preferences for a mobile application to support management of anxiety and depression. However, we observed generational differences in using social media as a source of support. Younger participants (less than 50 years old) were more likely to voice that social media was used as a source of social support. For example, one 22-year-old participant stated:

“I think that in the age of social media now, if you tweet things, I guess, or make a post, then I guess that makes you feel less alone because you always realize that other people agree or go through the same things.” [Speaker 4, Group 1, 22 years old]

A 36-year-old participant voiced:

“I think that also is leading to a change generationally, what we do to take care of our mental health…I've met people on Twitter that I've traveled with that I did not know otherwise, Black women in particular…or on the phone for three hours, or making arrangements to meet at conferences, or we're in the same place or something like that. So, I feel like that generational leap there, I feel like there is a shift in what's happening with how Black women are dealing with mental health and it feels like maybe you don't have to take on the whole world.” [Speaker 2, Group 1, 36 years old]

Alternatively, a 63-year-old participant shared:

“I'm not into all that social media stuff. I don't know about people that are not into social media, I guess, we sometimes think that what the young people put out there is not appropriate.” [Speaker 3, Group 1, 63 years old]

## Discussion

### Principal findings

The main findings of the study informed priorities for design of a mobile app to support management of anxiety and depression among Black women. Desired content was either informational (e.g., information to find a Black woman therapist) or inspirational (e.g., encouraging stories about overcoming adversity). Suggested features allowed users to monitor their progress, practice healthy coping techniques, and connect with others. Participants also stressed the importance of feeling “a sense of community” while using the app. Also, transparency about who created and owns the app, and how users' data will be used and protected was recommended to establish trust. There is a paucity of peer-reviewed literature on interventions that used culturally-informed telehealth modalities to manage anxiety and depression among Black American adults, irrespective of physical health conditions (eg, HIV positive) or special circumstances (eg, caregiver of patient with dementia) (15). However, the recommendations produced by this study are consistent with those found in previous literature which highlights the need for educational, psychotherapy, and personal development components in a mental health app designed to help users manage anxiety and depression (16, 17).

A study by Sarkar and colleagues investigated the usability of commercially available apps for depression and found that “while patients express interest in using technologies for self-management, current tools are not consistently usable for diverse patients” (18). The results of this study supported the need for culturally tailored mental health apps. Some of the study participants used mental wellness apps in the past, however, engagement was short-lived due to lack of culturally relevant content, feeling that the app was not working for them, or limited access to content and features (e.g., free trial ending). To increase adoption and daily use of a mental health app participants voiced that the app must promote a sense of community. A recent literature review found that incorporating mechanisms for peer support in mental health interventions has been effective (19). Previous studies have found, in general, that when individuals with a mental health condition connect through social platforms with people going through similar experiences, it gives them a sense of belonging, promotes self-efficacy, and encourages help-seeking behaviors (20). Digital communities can also help to challenge stigma and provide emotional support (21).

Furthermore, social support is important to the well-being of Black women, however, self-help coping can overcome the impact of a lack of social support on well-being (22). Creating an app with both peer support and opportunities to learn healthy coping skills and psychoeducation for mood management is suggested. The inclusion of preferred content and features, such as group chat rooms for peer support, will help researchers and developers create apps that are both useful and “sticky” (23). A sense of community is known as a protective factor. A protective factor moderates the effect of a vulnerability or risk factor on development, promoting adaptive development and resilience, or simply the capacity for positive outcomes despite challenging circumstances (24). Increasing overall sense of community within the app could mitigate risk factors that are connected to symptoms of depression and anxiety.

Acceptance of a mental health app is hindered by lack of transparency about who owns that app, and how users’ data will be used and protected. A major concern of focus group participants was that the data collected about their mental health would be used to harm them personally or Black women in general. Recent news of data breaches at credit bureaus and social media companies has diminished peoples' confidence that their private data will be protected when using an app. Concerns around privacy and confidentiality must be addressed for successful implementation of mHealth interventions for Black women (9). Clear communication on how users' data will be used and protected should be disclosed. Trust can only be earned through transparency and consistency in being good stewards of the data that we have been entrusted with.

Regardless of the mode of delivery, these insights will help future researchers and clinicians identify key topics that should be covered when considering content to include in mental health interventions for Black women. Furthermore, the findings will provide a better understanding of the concerns and limitations around using mHealth modalities to deliver mental health services and resources to Black women. One caveat is that the use of apps to receive mental health support may not be appropriate for everyone. If an app is used as part of a treatment plan, clients should be screened to determine if the use of this modality is appropriate (25).

By centering the needs and perspectives of Black women in the design of a mental health app, this research contributes to the growing body of work that aims to extend the reach of consumer health informatics technologies among groups that are underserved (26, 27). Incorporating the user-design requirements of participants may help them become more engaged and empowered in regards to their mental health (28). The design preferences of the study participants confirm the importance of incorporating trust-centered design components (29) and designing health information technologies for stigma reduction (30), networked empowerment (31), and social support exchange (32). Designing a mental health app for and with Black women may help to advance digital health equity by providing a tool that addresses their specific needs and preferences.

### Limitations

While participants in the focus groups shared common thoughts, recommendations, and opinions which are representative of their experiences, the findings should not be generalized to all Black women. One of the main limitations was that most of the focus group participants were under 50 years old, therefore, the sample skewed towards capturing the thoughts and opinions of younger Black women. These factors may limit the generalizability of the findings to older Black women. We did not collect income data to determine socioeconomic status; however, most participants obtained a Bachelor's degree or higher (95%). Furthermore, due to the geographical restriction in recruiting participants, the results may not reflect the opinions and perceptions of a nationally representative sample. Given the stigma of mental illness in the Black community, participants may also have felt peer pressure to give socially desirable answers to the moderator's questions. These limitations can be mitigated in the future by adding one-on-one structured interviews with participants in order to reduce social pressure and potential “groupthink”. Despite these limitations, the study results yielded useful information that will help researchers to understand the mental health needs of Black women and provide recommendations for developing a mobile app to help this population manage anxiety and depression.

### Conclusions and future directions

The findings of this study provide recommendations for the design of a mobile app supporting management of anxiety and depression symptoms among Black women. The user-centered recommendations may serve as a starting point when developing mental health apps for women from other racial/ethnic groups. However, when developing digital interventions, the intended users should inform the design so that the final product includes content and features that address their specific needs and interests. This user-centered design approach may be adopted to develop consumer health informatics technologies that aim to address the adverse health outcomes Black American women experience resulting from anxiety and depression (i.e., obesity, cardiovascular disease, suicide) (33–38). Furthermore, addressing the intersections between mental health, race, and gender should be evaluated in order to enhance the applicability of the app within minoritized communities. Gaining more insight into their lived experiences is essential to develop content that is not only clinically sound but also culturally responsive. We should focus on promoting mental wellness in community settings. Efforts must be made to include individuals that are not connected to care, and to provide them with the appropriate screenings and culturally relevant tools to support their mental health.

**Figure 2:**
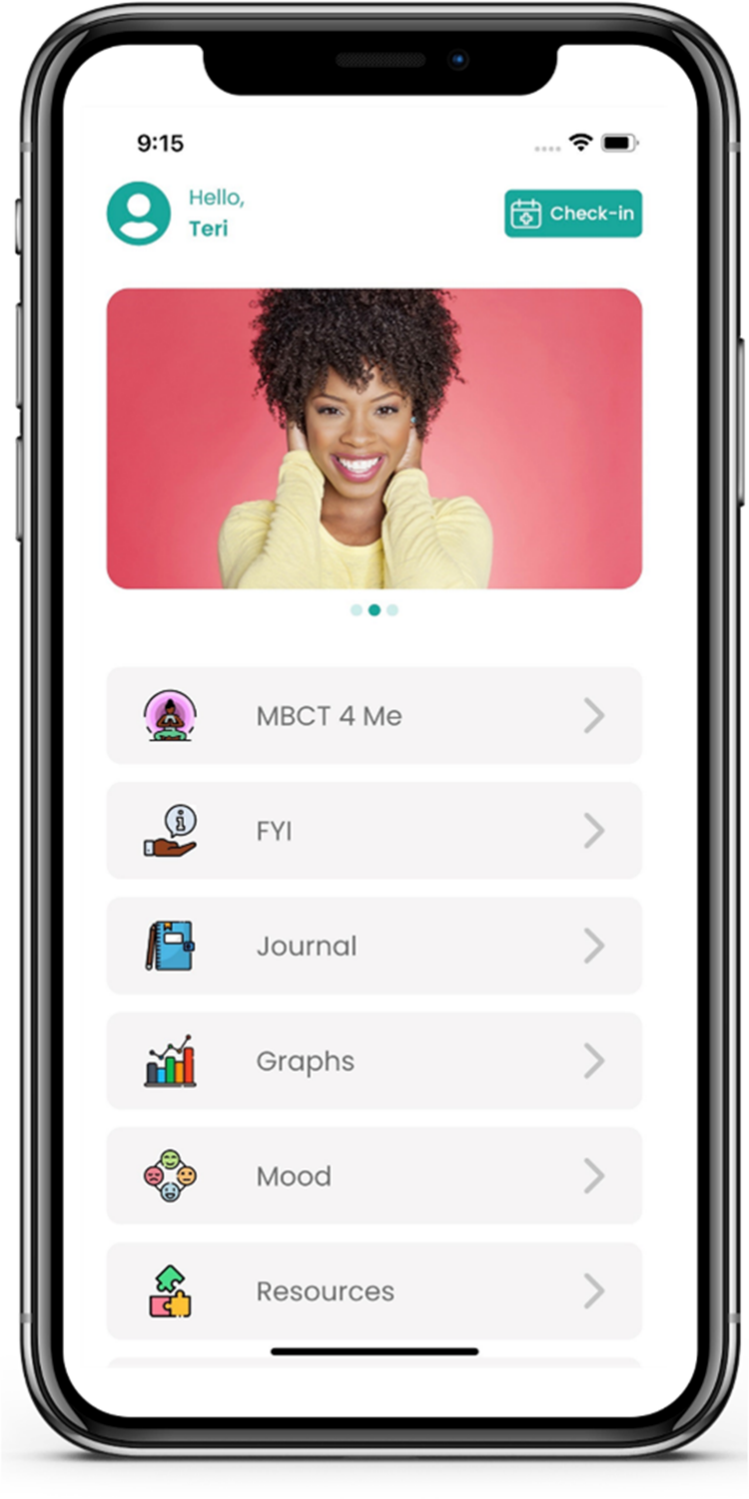
Mental health mobile app home screen.

Based on feedback from the focus groups, an initial prototype of an app designed to support management of anxiety and depressive symptoms among Black women was developed and usability testing was conducted (39). The initial prototype included a guided thought journal; information about anxiety and depression (including facts about the prevalence of anxiety and depression among Black women); self-assessments for depression (40) and anxiety (41); mood rating; graphs to track trends in depression and anxiety severity, and mood rating history; culturally-informed resources (e.g., links to the Therapy for Black Girls therapist directory and podcast); and a self-care planner. Future work includes continuing to incorporate the user-centered recommendations from the focus groups, including therapeutic content such as mindfulness-based cognitive therapy, into the app. Figure 2 shows the home screen for the current version of the app. The rise in use of virtual platforms for delivery of health care since the COVID-19 pandemic has been a catalyst for the development of new digital health tools, such as mental health apps. However, mental health apps should be culturally responsive and created in partnership with intended users (42, 43) to increase positive impact and engagement.

## Data Availability

The raw data supporting the conclusions of this article will be made available by the authors, without undue reservation.

## Appendix A

### mHealth for mental health app development for Black American women focus group interview guide

#### Part I

Let's start the discussion by talking about how you define mental health.

1. When you think about “good mental health,” what goes through your mind?
2. When you think about “bad mental health,” what goes through your mind?
3. What, if anything, do Black women do to maintain good mental health?
4. Are there certain qualities that Black women have that help us maintain “good” mental health? If so, what are they?
5. Are there things that Black women deal with that other groups might not have to deal with?
  a. How do you think that affects our mental health? Can you talk more about that?
6. What are some of the things in the past that have caused you to feel anxious?
  a. How did you deal with them?
  b. What type of support or resources would have been helpful to have access to during that time?
7. What are some of the things in the past that have caused you to feel depressed?
  a. How did you deal with them?
  b. What type of support or resources would have been helpful to have access to during that time?
8. How do you feel about using mental health services, such as seeing a therapist?
  a. Do you think that there's a stigma around using this kind of care? Can you tell me more about that?
  b. Do you feel that the stigma around using mental health services has changed in the last five years? Can you tell me more about that?
9. Before we switch gears to talk about the app, is there something else you’d like to discuss that I haven’t asked you?

#### Part II

The last half of our session will be used to ask questions about your use of mobile apps, and what information, resources, and features you would like in a smartphone app designed to help Black women manage anxiety and depression.

1. Do you currently use mental health and wellness apps?
  a. Which apps do you use?
  b. Which features do you like most?
  c. Which features do you like least?
2. What topics would you like to see addressed in the app?
  a. Are there any topics that you think would be particularly helpful for Black women?
3. If you could design an app to help Black women manage anxiety and depression, what kinds of features would it have?
  a. Are there any specific activities you would like to be able to complete in the app?
  b. Would you like to be able to track your emotions? Can you tell me more about that?
  c. What types of notifications would be helpful?
    i. How often would you like to receive them?
  d. Would you like to be able to connect with a mental health professional through the app?
    i. How would you like to connect with them?
4. What type of support or resources would be helpful to have information about in the app?
5. What, if anything, would make you want to use it regularly?
6. What would get in the way of you using it regularly?
7. Do you have any concerns about using a mental health app?
8. If you were designing the app, how would you get the word out about the app?
